# Reviewing the role of gut microbiota in the pathogenesis of depression and exploring new therapeutic options

**DOI:** 10.3389/fnins.2022.1029495

**Published:** 2022-12-08

**Authors:** Wenjie Han, Na Wang, Mengzhen Han, Meng Ban, Tao Sun, Junnan Xu

**Affiliations:** ^1^Department of Breast Medicine, Liaoning Cancer Hospital, Cancer Hospital of China Medical University, Shenyang, China; ^2^Department of Pharmacology, Liaoning Cancer Hospital, Cancer Hospital of China Medical University, Shenyang, China; ^3^Liaoning Microhealth Biotechnology Co., Ltd., Shenyang, China; ^4^Department of Breast Medicine, Cancer Hospital of Dalian University of Technology, Liaoning Cancer Hospital, Shenyang, China

**Keywords:** major depressive disorder (MDD), microbiota-gut-brain axis, metabolite, inflammation, neuroendocrine, gut microbiota

## Abstract

The relationship between gut microbiota (GM) and mental health is one of the focuses of psychobiology research. In recent years, the microbial-gut-brain axis (MGBA) concept has gradually formed about this bidirectional communication between gut and brain. But how the GM is involved in regulating brain function and how they affect emotional disorders these mechanisms are tenuous and limited to animal research, and often controversial. Therefore, in this review, we attempt to summarize and categorize the latest advances in current research on the mechanisms of GM and depression to provide valid information for future diagnoses and therapy of mental disorders. Finally, we introduced some antidepressant regimens that can help restore gut dysbiosis, including classic antidepressants, Chinese materia medica (CMM), diet, and exogenous strains. These studies provide further insight into GM’s role and potential pathways in emotion-related diseases, which holds essential possible clinical outcomes for people with depression or related psychiatric disorders. Future research should focus on clarifying the causal role of GM in disease and developing microbial targets, applying these findings to the prevention and treatment of depression.

## Introduction

As early as 1880, Kraepelin established major depressive disorder (MDD) as an independent mental disorder (Kendler, 2017). In addition to some physiological symptoms like weariness and sleep problems, it primarily distinguishes high neuroticism, low self-esteem, severe negative and repetitive thinking, and impaired cognitive reactivity. In more severe cases, delusions and suicidal conduct may also manifest (Struijs et al., 2021). In 2019, the World Health Organization estimated that the global economic cost of anxiety and depression disorders was up to $1 trillion, and this trend expects to rise (Doran and Kinchin, 2019). Although there has been a revolution regarding psychotropic treatments and psychotherapeutics in the last few decades, one-third of patients still do not achieve sustained remission during an MDD episode (Monroe and Harkness, 2022; Rush et al., 2022). Therefore, there is an urgent need to gain new insights into these psychiatric disorders’ underlying pathological mechanisms to help patients alleviate symptoms and improve their quality of life.

The pathogenic factors of MDD are also very complex, including biological, psychological, and social factors (Rostila, 2020; Dudek et al., 2021). Several hypotheses also exist for the pathogenesis of depression, but most of them have some limitations. For example, the most widely accepted theory is the monoamine hypothesis. It argues that insufficient activity of monoaminergic neurons causes depression (Perez-Caballero et al., 2019). Recent clinical findings have demonstrated that up to 30% of individuals do not respond to the monoamine hypothesis class of medications. Moreover, this hypothesis does not explain the response latency of antidepressants (Boku et al., 2018). Subsequently, receptor theory claimed that adaptive changes in receptors could explain this gradual clinical response (Wang H.-Q. et al., 2021). This theory claims that abnormal signal transmission causes abnormal neurotransmitter receptor coupling or changes in downstream signaling cascade responses (Jesulola et al., 2018). However, the durability and safety of drugs targeting receptor therapy (e.g., ketamine) and differences in gender expression of receptors add to the limitations of current treatment (Ma et al., 2018; Rosenblat et al., 2019). This difference in expression brings attention to the genetic level. For the genetic hypothesis, there is much epidemiological evidence for a genetic contribution to MDD susceptibility (Mitchell et al., 2022). Many genes, such as NEGR1, CTC-467M3.3, TMEM106B, etc., have a strong, colocalized and potentially causal association with depression (Dall’Aglio et al., 2021). But it was found that different definitions of depression have distinct genes, which makes it necessary to be more cautious in our research on the genetic studies (Wu et al., 2022). From our literature review, there is no one-size-fits-all statement about the pathophysiological mechanisms of depression. The most likely explanation is that it involves multiple mechanisms and that these mechanisms are interrelated, ultimately manifesting in the human body in a range of pathological symptoms.

Over the past few decades, with advances in sequencing technology, researchers have developed a more profound understanding of the human microbiome. It has also shown that symbiotic microorganisms in the body, especially GM, are involved in regulating the balance between health and disease (Cani, 2018; Sorbara and Pamer, 2022). The latest hypothesis for depression has also evolved into the concept of the MGBA (Chang et al., 2022). This formulation clarifies the existence of a bidirectional signaling network between the brain and the gut and interconnects neural, metabolic, and immune regulatory mechanisms (Tremblay et al., 2021). MDD is also no longer considered to be a single mental condition, according to contemporary psychological and bioinformatics theories. It has obvious biological manifestations, mainly involving four aspects: the dysfunction of the brain, the immune system, the hypothalamus–pituitary–adrenal (HPA) axis, the immune system, and gut-brain dysfunction (Liang et al., 2018). Brain dysfunction mainly manifests as neurotransmitter imbalance (Dalvi-Garcia et al., 2021), impaired neuroplasticity (Tartt et al., 2022), and abnormal neurogenesis (Kim and Park, 2021). Immune system disorders mainly reflect oxidative stress and chronic inflammation (Czarny et al., 2018; Luca et al., 2019). The primary sign of HPA axis dysfunction is the maladjustment of negative feedback mechanisms (Du et al., 2020; Cheiran Pereira et al., 2021). Gut dysbiosis and accompanying gastrointestinal diseases are the main symptoms of gut-brain dysfunction (Oungpasuk et al., 2020; McGuinness et al., 2022). The existing literature suggests that the GM is directly or indirectly involved in the above processes through the MGBA, which is a good inspiration for us to carry out mechanistic studies or search for microbial biomarkers (Barrio et al., 2022; Suda and Matsuda, 2022). This paper reviews the possible mechanisms involved in the pathogenesis of MDD by GM based on some preclinical and clinical studies and discusses potential therapeutic ideas based on regulation of GM, which may provide some reference for future research on the mechanisms of depression prevention, etiology, and treatment.

## Characteristic gut microbiota profile in depressive states

Although significant challenges remain in studying host-microbiota interactions, much metabolites-focused research has identified some microbial targets associated with disease progression, including metabolic disease, cardiovascular disease, psychiatric disorders, and others (Wang and Zhao, 2018; Wu et al., 2021). In terms of depression, there is also substantial evidence supporting the critical regulatory role of GM and its metabolites in disease progression (Table 1). Therefore, targeting the GM profile may be an effective strategy to facilitate depression-related pathological features.

**TABLE 1:** Studies investigating the biological link of microbial metabolites on depressive-like behavior.

Family	Microbial metabolite	Host behavior outcomes	Molecular mechanisms	Strains	Stimulation	Model	References
Neurotransmitter	5-HT	↑Turotransmitt ↓depressive-like behaviors	↓5-HT in the colonic; ↑5-HT in the frontal cortex and hippocampus	*Lactobacillus rhamnosus*	CUMS	Mice	Li H. et al., 2019
		↓Anxiety and depressive-like behavior	↑5-HT in the hippocampus, PFC, and striatum	*Lactobacillus paracasei PS23* (live)	Corticosterone-induced	Mice	Wei et al., 2019
	DA	↓Anxiety and depressive-like behavior; altered the GM structure	↑ DA and 5-HT in the striatum	*Lactobacillus plantarum*	GF	Mice	Liu W.-H. et al., 2016; Dhaliwal et al., 2018
		↓Depressive-like behaviors; restoration of the GM structure	Restored abnormal variations in DA, corticosterone, and BDNF	*Akkermansia muciniphila*	CRS	Mice	Ding et al., 2021
		↓Stress response of the HPA axis; ↓IFN-γ; ↓reversed gut dysbiosis; depressive-like behaviors	↓DA in the hypothalamus	*Bifidobacterium CECT 7765*	MS	Mice	Moya-Pérez et al., 2017
	γ-GABA	↓Depressive-like behavior	↑GABA	*Lactobacillus plantarum 90sk*; *Bifidobacterium adolescentis 150*	FST	Mice	Yunes et al., 2020
		↓Corticosterone; ↓anxiety and depressive-like behavior	↓GABA (Aα2) mRNA expression in the PFC and amygdala	*Lactobacillus rhamnosus*	Stress-induced	Mice	Bravo et al., 2011
	NE	Normalization of the immune response; reversal of behavioral deficits	↑NE in the brainstem; ↓IL-6	*Bifidobacterium infantis*	FST	Mice	Desbonnet et al., 2010
	Glu	↓Anxiety and depressive-like behavior	Differential regulation of Glu receptors and MicroRNA124a/132	*Lactobacillus paracasei*	SSE	Mice	Karen et al., 2021
SCFAs	Sodium butyrate	↓Hippocampal microglial activation; ↓depressive-like behavior	Recovery of the microglial gene profiles	*L. plantarum, B. infantis, Clostridium butyricum and F. prausnitzii*	LPS-induced	Mice	Yamawaki et al., 2018
		↓Behavioral deficits; recovery of BBB impairments	↑5-HT; ↑TJs protein levels; ↑BDNF expression in the hippocampus		CUMS	Mice	Sun et al., 2016
		↓Neuroinflammatory; ↓depressive-like behavior	↓IL-1β; ↓ IL-6; ↓TNF-α		LPS-induced	Mice	Qiu et al., 2020
	Sodium Propionate	↓Depressive-like behavior	Reduction of catabolism of NE, tryptophan, and DA	*Phascolarctobacterium succinatutens; Veillonella* spp.*; Megasphaera elsdenii*	CUMS	Mice	Louis and Flint, 2017; Li et al., 2018
Tryptophan metabolites	Indoles	↓Motor activity; vagus nerve activation; ↑helplessness; ↑anxiety and mood disorders	↑c-Fos protein expression in the dorsal vagal	*Escherichia coli*	GF	Mice	Jaglin et al., 2018
	Indole derivatives	Positive association with recurrent depressive symptoms	↑3-indoxyl sulfate	gut dysbiosis		Human cases	Philippe et al., 2021
Other metabolites	Lactate	↓Immobility time in the open space; ↓depressive-like behavior	↓GSK3α/β in the hippocampus; ↓COX-2 mRNA in the hippocampus; ↓expression of PDE4D and NOS1	*L. lactis, L. gasseri, L. reuteri, Bifidobacteria, and Proteobacteria* (Louis and Flint, 2017)	Corticosterone-induced	Mice	Carrard et al., 2018
		↑Social avoidance; ↓anxiety and depressive-like behavior	Hippocampal class II HDAC5 deactivation		CSDS	Mice	Karnib et al., 2019
	Bile acids	↓Depressive-like behavior	↑BDNF expression in hippocampal	*Lactobacilli*, *bifidobacteria*, *Clostridium* and *Bacteroides* (Wahlström et al., 2016)	CUMS	Mice	Chen et al., 2018
		↓Neuroinflammation; ↓oxido-nitrosative; ↓depressive-like behavior	↓TNF-α and IL-6 in the hippocampus and PFC		CUS	Mice	Lu et al., 2018
	Choline metabolites	↓Depressive-like behavior	↑DNA methylation; ↑expression of the insulin receptor	The order *Clostridiales*, the genus *Ruminococcus*, and the taxon *Lachnospiraceae* (Wang et al., 2015)	MS	Mice	Paternain et al., 2016
	Vitamins (folate)	↓Cardiovascular measures; anxiety and depressive-like behavior	↓DNA methylation in the hippocampus; ↑brain methionine levels	*Lactobacillus* and *Bifidobacterium* (Gu and Li, 2016)	MS	Mice	McCoy et al., 2016

5-HT, 5-Hydroxytryptamine; PFC, prefrontal cortex; DA, dopamine; γ-GABA, Gamma-aminobutyric acid; NE, Norepinephrine; Glu, Glutamate; GM, gut microbiota; CUMS, chronic unpredictable mild stress; GF, germ-free; BDNF, brain-derived neurotrophic factor; CRS, chronic restraint stress; HPA, hypothalamic-pituitary-adrenal; IFN-γ, interferon-gamma; MS, maternal separation; FST, Forced-Swim Test; IL-6, interleukin-6; SSE, stressful social experience; LPS, lipopolysaccharides; BBB, blood-brain barrier; TNF-α, tumor necrosis factor-α; GSK3α/β, Glycogen synthase kinase 3 alpha/beta; COX-2, Cyclooxygenase 2; PDE4D, Camp-Specific 3′,5′-Cyclic Phosphodiesterase 4D; NOS1, Nitric Oxide Synthase 1; HDAC5, Histone Deacetylase 5; CSDS, Chronic Social Defeat Stress; CUS, Chronic Unpredictable Stress; ↑, upregulated; ↓, downregulated.

To explore the characteristic GM composition, many scientists began to take different approaches to establish rodent depression models to observe variations. For example, olfactory bulbectomy (OB), chronic unpredictable mild stress (CUMS), chronic forced swim test, social defeat stress, learned helplessness, etc. (Ramaker and Dulawa, 2017). Correspondingly, we have observed the characteristic changes in these different depressed mouse models (Medina-Rodriguez et al., 2020; Kosuge et al., 2021; Jiang X. et al., 2022; Meng et al., 2022). For example, mice exposed to CUMS exhibited a lower abundance of *Lactobacillus* and a relatively higher level of *Akkermansia* (Li N. et al., 2019). Park et al. (2013) found that in addition to gut dysbiosis in OB mice, signaling pathways projecting from the prefrontal cortex (PFC) to the hypothalamus were also impaired. These studies suggest that signals of depression can also be transmitted from top to down.

In addition, some clinical evidence points to the characteristic GM composition of depression (Lai et al., 2021; Zhong et al., 2022). In April 2022, McGuinness et al. (2022) conducted a systematic review of the community composition between 1,038 MDD cases and 1,048 non-MDD controls. In the aspect of species composition, butyrate-producing bacteria significantly reduced in the MDD group, such as *Faecalibacterium* and *Coprococcus* (Valles-Colomer et al., 2019). The higher levels of bacteria taxa are mainly associated with the production of lactic acid, glutamate metabolism, and GABA metabolisms, such as *Lactobacillus*, *Enterococcus*, and *Streptococcus* (Lyu et al., 2018; Yogeswara et al., 2020). Although the observations varied considerably between studies at the genus level, the same trends were shown at the phylum and class levels, indicating that there may be some confounding factors in sample selection. In terms of diversity, some studies have come up with various opinions. For example, Bai et al. (2021) suggested that there was no significant difference in alpha diversity (species richness) between MDD patients and healthy controls (Chao, *p* = 0.62). In contrast, Rong et al. (2019) indicated a significant difference between the two groups, with the MDD group being significantly lower (Chao, *p* = 0.003). Intriguingly, the populations in both studies were from China, excluding heterogeneity due to region. Recent findings in other psychiatric disorders, such as Parkinson’s disease and autism spectrum disorder, have also reported equivocal alpha diversity findings (Ho et al., 2020; Nuzum et al., 2020). Clearly, alpha diversity is not an appropriate indicator of the mental health status of the host.

## Potential mechanism of gut microbiota involved in the regulation of major depressive disorder

**FIGURE 1:**
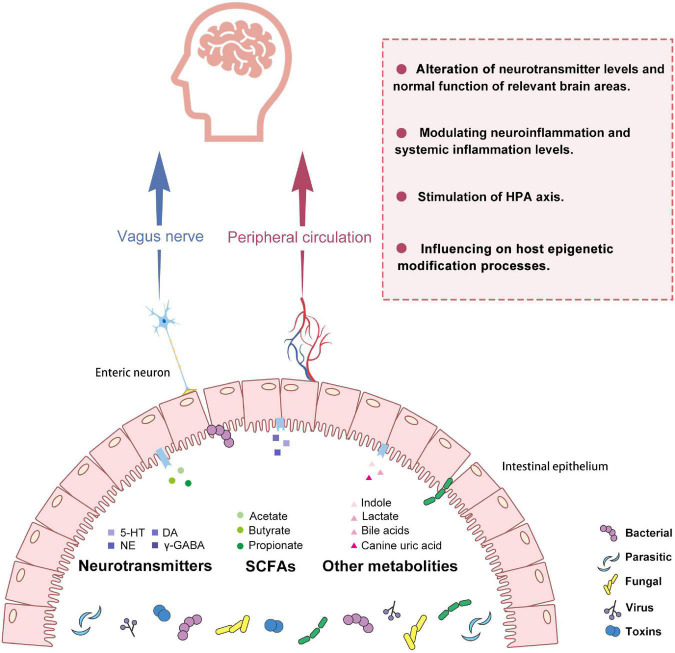
Potential pathways for gut microbiota to make connections with the brain in depression. GM and its metabolites include SCFAs, neurotransmitters, etc., which can act as a bridge to transmit information from the gut to the brain. This communication consists mainly of neural and humoral pathways. On the one hand, it can affect neuroimmune and neuroendocrine mechanisms closely related to the vagus nerve. On the other hand, GM and its metabolites can also cross the intestinal barrier and enter the systemic circulation to modulate host neurotransmitter levels, the immune system, the endocrine system, and epigenetic processes. GM, gut microbiota; 5HT, 5-hydroxytryptamine; DA, Dopamine; NE, Norepinephrine; γ-GABA, Gamma-aminobutyric acid; SCFAs, short-chain fatty acids; HPA, hypothalamic-pituitary-adrenal axis.

Based on the strong correlation between GM and depression, in this section, we describe possible mechanisms by which GM mediates the regulation of MDD. These mechanisms rely mainly on the vagus nerve and peripheral circulation for signaling. Peripheral circulation primarily interferes with intracerebral neurotransmitters, inflammation, HPA axis reactivity, and epigenetic modification processes (Figure 1).

### Reliance on the vagus nerve for signaling

In the intestine, the enteric nervous system, which consists of numerous enteric neurons, can independently regulate many complex functions (Rao and Gershon, 2016; Li et al., 2020). Enteric neurons locate beneath the intestinal epithelium. They can communicate directly or indirectly with GM, eventually transmitting signals to other body organs (including the CNS) (Cawthon and de La Serre, 2018). Yang et al. (2022) found that severing the subdiaphragmatic vagus nerve could reduce depression-like phenotypes in the depression model mice and had effects on the composition of the GM. Clearly, the subphrenic vagus nerve is one of the pathways of depression signaling. Zhang et al. (2020), Pu et al. (2021), and Wang S. et al. (2021) obtained depressed mouse models by knocking out different genes to perform experiments as above, all of which all confirmed the existence of this pathway. In addition, it has been found that the antidepressant effect of some probiotics (*Lactobacillus intestinalis* and *Lactobacillus reuteri*) also depends on the integrity of the vagus nerve (Wang et al., 2020). These studies demonstrate the importance of vagal pathways in depression, but the molecular mechanisms of how GM affects the enteric vagus need to be explored in the future.

### Influence on neurotransmitter levels and function of relevant areas

The monoamine hypothesis has focused scientists’ attention on monoamine neurotransmitters such as 5-Hydroxytryptamine (5-HT), dopamine (DA), gamma-aminobutyric acid (γ-GABA), noradrenaline (NE), etc.(Jiang Y. et al., 2022). Some specific genera like *Candida*, *Streptococcus*, and *Escherichia* can directly produce 5-HT to affect peripheral and cerebral 5-HT levels. Under normal physiological conditions, 5-HT cannot pass through the blood-brain barrier (BBB), but under pathological conditions, tryptophan produced by GM can penetrate the impaired BBB through blood circulation. And then, it produces 5-HT precursors in the brain to supplement the 5-HT deficiency in the brain (Makris et al., 2021). Pleasure deficiency is also associated with the dysfunction of the DA system (Rincon-Cortes and Grace, 2020). Tsavkelova et al. (2000) detected the neurotransmitter of microorganisms by high-performance liquid chromatography and found that *Bacillus subtilis*, *Escherichia coli*, etc., could produce DA. The administration of *Bifidobacterium pseudocatenulatum* CECT 7765 has been shown that it can decrease the intestinal hypercatecholaminergic activity of the depressed model and reduce intestinal DA concentrations, ultimately attenuating the excessive stress response of the HPA axis (Moya-Perez et al., 2017; Huang and Wu, 2021). In terms of γ-GABA, it acts as an inhibitory transmitter in regulating multiple functions in the brain, including anxiety, motivation, and the reward system (Petty et al., 1995; Lowes et al., 2021; Zhang et al., 2021). *Lactobacillus plantarum* and *Bifidobacterium adolescentis*, ascertained as the most effective GABA-producing bacteria, have antidepressant effects comparable to fluoxetine in mice models (Yunes et al., 2020). In the case of NE, animal studies have shown that *Bifidobacterium infantis* restores basal NE concentrations in the brainstem and reverses behavioral deficits in depressed mice through the humoral route (Desbonnet et al., 2010). Although many studies have demonstrated the close relationship between GM and these neuroactive metabolites, experimental results have mostly been limited to animal studies. The reproducibility of these mechanisms in clinical studies still needs to be invested in a more in-depth study.

Patients with MDD exhibit dysfunction or hisomorphic disruption in the brain associated with emotions, such as abnormal neurogenesis (Kim and Park, 2021), targeted disruption of the BBB (Dion-Albert et al., 2022), abnormal amygdala and hippocampus function (Price and Duman, 2020; Grogans et al., 2022), impaired structure and function of glial cells (Zhou et al., 2021). Scientists are also investigating the effects between GM and brain-related tissues. The colonization of *Bacteroides fragilis*, *Bacteroides uniformis*, has been shown to impair hippocampal neurogenesis and deplete serotonin levels in the brain, exacerbating depressive states (Zhang et al., 2022). GM diversity and composition also affect the release of gut peptides, including cholecystokinin, glucagon-like peptides, and so on. These gut peptides cross the damaged BBB and directly influence the hypothalamic arcuate nucleus, ultimately affecting the normal function of the amygdala and nucleus accumbens, which is associated with mood (Weltens et al., 2018; Merchak and Gaultier, 2020).

### Modulating inflammation levels

Clinical studies have shown that MDD patients have a higher serum concentration of IL-1β, IL-4, IL-8, and IL-10. The level of inflammatory markers is also closely related to the severity of depression (Ogłodek, 2022). The close association between inflammation (neuroinflammation and systemic inflammation) and MDD has also been the subject of many in-depth studies (Hayley et al., 2021; Zheng et al., 2021).

In terms of neuroinflammation, the current view focuses more on central microglia (Jia et al., 2021). Some forms of depression are sometimes called microgliopathy (Deng et al., 2020). Erny et al. (2015) observed that microglia in germ-free mice are severely defective and their immune system disorder. Colonization with a complex GM to mice can restore microglia features (Erny et al., 2015). This proves that GM is also involved in shaping the resident immune system in the CNS. In turn, neuroinflammation can affect the normal function of the GM. The inflammatory environment can induce the tryptophan metabolism of the microorganism to be biased toward the kynurenine pathway. And it eventually metabolized to quinolinic acid (Ryan et al., 2020). Neurotoxic quinolinic acid can affect the function of astrocytes and induce depressive symptoms (Öztürk et al., 2021). Significantly, GM can also positively modulate the brain’s inflammation level (Merchak and Gaultier, 2020). The short-chain fatty acids (SCFAs) can be produced by *Eubacterium rectale*, *Roseburia faecis, Eubacterium hallii*, etc. It can cross the BBB and act on the aryl hydrocarbon receptors. They are involved in the systemic regulation of cytokine profiles and reduce neuroinflammation (Ortega et al., 2022). Indole derivatives may also play a neuroprotective role by reducing neuroinflammatory markers through activating astrocytes (Rothhammer et al., 2016). For example, *Bifidobacterium* spp. and *Lactobacillus* spp. can produce indole-related compounds, Indole-3-lactic acid, and indole-3-aldehyde, which may be related to their antidepressant effects (Roager and Licht, 2018).

How does peripheral inflammation affect the level of intracerebral cytokine levels? There is much evidence that MDD patients have symptoms of increased intestinal permeability (Akdis, 2021). Ohlsson et al. (2019) observed a statistically significant correlation between intestinal permeability markers and inflammatory factors in patients with depression. Alvarez-Mon et al. (2020) continued investigating this correlation and found that bacterial translocation blunted the expansion of regulatory T lymphocytes, thus amplifying the tendency for inflammation. Intestinal symbiont translocation can also drive increased nitrosation to activate immune-inflammatory and oxidative pathways. They negatively affect neurogenesis and synaptic plasticity, ultimately inducing depression (Maes et al., 2019). Increased intestinal permeability further increases the plasma concentration of lipopolysaccharide (LPS). It is also known as endotoxin, cell wall components of Gram-negative bacteria (Kuti et al., 2020). LPS can directly destroy BBB function (Peng et al., 2021). Meanwhile, it can also stimulate the Nuclear factor-k-gene binding pathway and promote cytokine production through binding to Toll-like receptor 4 (Guo et al., 2019). After BBB injury, a large number of cytokines and neurotoxins will enter the brain and further activate the inflammatory response and damage the brain tissue. Moreover, stress can also induce BBB dysfunction (Matsuno et al., 2022). This vicious cycle eventually leads to the onset of depression. Therefore, we believe that some harmful bacteria open channels for immune factors by disrupting the intestinal mucosal barrier and the BBB, which could explain the immune disorders present in the brain of most depressed patients.

### Stimulation of hypothalamus–pituitary–adrenal axis

The HPA axis is an essential component of the MGBA and regulates the host stress response (Karaca et al., 2021). MDD patients also exhibit hyperactivity of the HPA axis. It is mainly in the form of increased glandular reactivity, disruption of hormone secretion, and negative feedback dysregulation (Mikulska et al., 2021). These disorders can further lead to intestinal inflammation, neuronal damage, and cortisol overproduction, all of which are associated with depression (Liu et al., 2020). Notably, adrenal glands in the HPA axis secrete cortisol with a circadian rhythm, which develops in parallel with the GM early in life (de Weerth, 2017). It can be seen that there is some complex connection between the GM and the HPA axis.

To date, many mechanisms have been proposed for how GM affects the HPA axis. First, as mentioned in the previous section, there is a close relationship between gut dysbiosis and inflammation. And these cytokines are also very effective activators of the HPA axis (Misiak et al., 2020). Proinflammatory cytokines can interfere with the cortisol cycle’s negative feedback to stimulate the HPA axis’ activity (Zhou et al., 2022). GM can also stimulate the HPA axis through other mediators capable of crossing the BBB, such as microbial antigens and prostaglandins (Mikulska et al., 2021). In addition to relying on these mediators, bacteria can directly participate in the intervention process. For example, LPS and peptidoglycan can activate the HPA axis by activating the innate immune system, which ultimately induces depression (Gonzalez-Santana and Diaz Heijtz, 2020; Smith et al., 2021). There is also evidence that GM can directly modulate steroidogenesis in the gut and adrenal to exaggerate HPA axis response (Vagnerová et al., 2019).

Second, GM-derived metabolites are also involved in regulating the HPA axis through humoral pathways. For instance, chronic indole (*Bacteroides* spp., *Lactobacillus* spp., and *Clostridium* spp.) can induce adrenomedullary Pnmt gene overexpression. And it can increase the catecholamine biosynthesis pathway in the adrenergic system along with noradrenaline. In this way, indole can increase the vulnerability of mice under chronic mild stress, ultimately leading to depressive behavior (Smith and Macfarlane, 1996; Park et al., 2013; Rothhammer et al., 2016; Qu et al., 2019). And beyond that, GM can also play a positive role in depression by modulating the HPA axis. For example, its downstream metabolites, SCFAs, can reduce gene expression of some encoding proteins in the HPA axis, thereby attenuating the stress response of the HPA axis (van de Wouw et al., 2018). Finally, GM can also influence the signal input to the sub-diaphragmatic vagus nerve. The nucleus of the solitary tract activates the HPA axis via noradrenergic neurons (Paton et al., 2000; Herman et al., 2016).

In fact, there is also evidence that the HPA axis also affects the GM in turn. In a study of irritable bowel syndrome, the HPA axis influences GM composition through vagal tone and cortisol homeostasis (Pellissier and Bonaz, 2017). While all of these mechanisms are strongly supported by these animal studies, there is a paucity of population studies. And there is a lack of longitudinal studies, so the causal relationship between the GM and HPA axis needs to be addressed in future studies.

### Alteration of epigenetic modifications process in the brain

Because of the heterogeneity of depression, its onset and progression are not well described by a single gene. On the contrary, it is more believed that the clinical manifestations of depression are co-presented by the activation or silencing of many genes caused by environmental factors (gut dysbiosis, diet, infection, etc.), the namely epigenetic mechanism (Alshaya, 2022). The current research mainly involves three pathways: post-translational histone modifications, RNA interference, and DNA methylation (Czarny et al., 2021).

Regarding histone modifications, related studies have pointed out that the acetylation site and the succinylation site on lysine in the hippocampus are close to depression (Liu et al., 2021b; Yu et al., 2021). GM can ease depression through this mechanism. Butyrate, a type of SCFAs, is a typical histone deacetylase inhibitor. It can promote histone acetylation to regulate the expression of proinflammatory factors and BDNF (Couto et al., 2020; Yang et al., 2020). For example, the administration of *F. prausnitzii* can increase butyrate levels in CUMS mice. The subsequent mechanism suggested that its antidepressant effect is closely related to the protein acetylation and the IL-6/STAT3/IL-17 pathway (Zhou et al., 2018; Hao et al., 2019). In addition, butyrate can also promote BNDF expression levels in the hippocampus. When treated with butyrate-producing bacteria (*Bifidobacterium infantis* and *Clostridium butyricum*), recovery of BDNF expression was observed in depressed mouse models (Sun et al., 2018; Tian et al., 2019). A multicenter clinical trial study also provided sufficient evidence (Kim et al., 2021). Moreover, related research shows that *Lactobacillus rhamnosus* can reduce hippocampal GABA_Aα 2_ mRNA expression in the depressive model (McVey Neufeld et al., 2019). However, whether this mechanism is also dependent on histone modification needs further evidence.

GM can also influence host mental health by affecting specific miRNA levels (Rosa et al., 2022). Liu et al. (2021a) examined relevant RNA levels in mice colonized with fecal from MDD patients. And they found that 200 mRNAs, 358 lncRNAs, and 4 miRNAs were differentially expressed compared to the control group. Functional analysis showed that these differential mRNAs were associated with inflammatory response (Liu et al., 2021a). Some probiotics, such as *Lactobacillus paracasei*, have also been shown to prevent early life stress-induced depression-like behavior by mediating differential regulation of microRNA124a/132 and glutamate receptors (Karen et al., 2021). Finally, affecting host DNA methylation is a necessary mechanism as well. In the study of Wei et al. (2014), sodium butyrate can up-regulate the expression level of TET1mRNA in the PFC of depressed mice, playing an antidepressant-like role. Subsequent mechanistic studies confirmed that this epigenetic effect is associated with TET1-mediated increased and decreased methylation of BDNF (Wei et al., 2014).

## Treatment of depression from the perspective of gut microbiota

**FIGURE 2:**
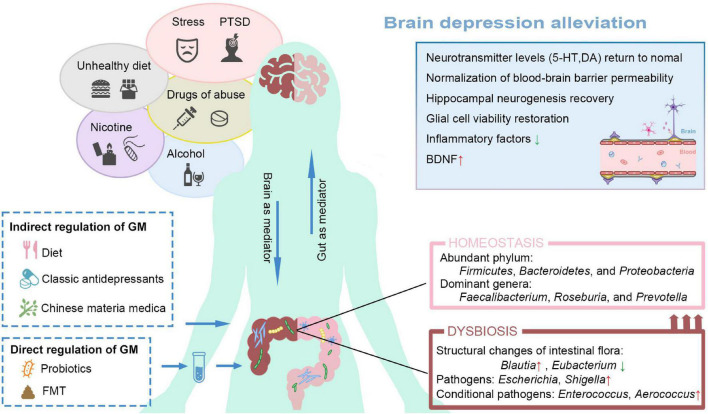
Ameliorates depression from the perspective of restructuring the GM. Poor lifestyle habits and external stressors can increase the risk of depression. MDD patients also exhibit gut dysbiosis. Restore intestinal homeostasis can be restored through direct or indirect regimens, thus alleviating depressive symptoms. GM, gut microbiota; 5HT, 5-hydroxytryptamine; DA, Dopamine; BDNF, brain-derived neurotrophic factor; PTSD, post-traumatic stress disorder; FMT, Fecal Microbiota Transplantation.

Based on the influential role of GM in the occurrence and pathogenesis of depression, we have noted that GM shows a powerful research potential in the study of antidepressant treatment, whether as an independent treatment or an auxiliary measure (Table 2). This section reviews some antidepressant approaches in regulating the GM structure, including classical antidepressants, CMM, dietary, and exogenous strains (Figure 2).

**TABLE 2:** Different antidepressant therapeutic approaches and their biological targets.

Intervention strategies	Drugs/strains	Biological targets	Effect on gut microbiota	References
Antidepressants	(R)-ketamine	NMDA receptor	Recovery of *Bacteroidales, Clostridiales*, and *Ruminococcaceae* levels.	Qu et al., 2017; Hua et al., 2022
	NMDEA	Inflammasome signaling pathway	Recovery of the richness and the dysbiosis among bacterial species.	An et al., 2020
	Rifaximin	Microglia	↑*Ruminococcaceae* and *Lachnospiraceae*	Li et al., 2021
Chinese materia medica	Jia Wei Xiao Yao San	Purine metabolism	↑*Lactobacillus animalis*	Ji et al., 2022
	Shihosogansan	NF-κB pathway	↓*Desulfovibrionaceae* and γ-*Proteobacteria*; ↑*Lactobacillaceae* and *Prevotellaceae*	Han et al., 2021
	Schisandra chinensis ameliorates	TLR4/NF-κB/IKKα	Alterations of the relative proportions of *Bacteroidetes* and *Firmicutes* phylum members	Yan et al., 2021
	Yang-Xin-Jie-Yu decoction	TCA cycle and propanoate metabolism	↑*Monoglobus*	Liang et al., 2022
	Xiaoyaosan	IL-6/MAPK1/STAT3/JUN	↑*Firmicutes*; ↓*Actinobacteria*	Liu et al., 2021c; Lv et al., 2021
	Kai-Xin-San	NGF, BDNF, and Trk receptor	↑*Allobaculum*, *Bifidobacterium*, and *Turicibacter*	Zhu et al., 2017; Cao et al., 2020
Dietary	Flavonoid-Rich Orange Juice	BDNF	↑*Lachnospiraceae* family	Park et al., 2020
	Okra (polysaccharide)	TLR4/NF-κB/MAPKs	↑*Firmicutes*; ↓*Bacteroidetes*, and *Actinobacteria*	Yan et al., 2020
	Almond	GPR43	Stimulating the growth of SCFAs-producing bacteria	Ren et al., 2020
Probiotics	*Lactobacillus reuteri*	IDO1	Restoring the *Lactobacillus* population	Marin et al., 2017
	*Bifidobacterium bifidum* BGN4 and *Bifidobacterium longum* BORI	BDNF	The relative abundances of *Eubacterium*, *Allisonella*, *Clostridiales*, and *Prevotellaceae* gradually changed.	Kim et al., 2021

NMDA, N-methyl-D-aspartic acid; NMDEA, N-(2-(7-methoxy-3,4-dihydroisoquinolin-1-yl)ethyl)acetamide hydrochloride; GABA, aminobutyric acid; TLR4, Toll-like receptor 4; NF-κB, nuclear factor kappa-B; IKKα, fwd 5′-ACCGTGAACATCCTCTG-3′, rev 5′-CTGCTCTGGTCCTCATT-3′; TCA cycle, tricarboxylic acid cycle; IL-6,interleukin-6; MAPK1, mitogen-activated protein kinase 1; STAT3, signal transducer and activator of transcription 3; JUN, transcription factor AP-1; NGF, nerve growth factor; BDNF, brain-derived neurotrophic factor; Trk, tyrosine kinase; SCFAs, short-chain fatty acids; GPR43, G protein-coupled receptor43; IDO1, indoleamine2,3-dioxygenase1; oleamine2,3 G pr↓, downregulated.

### Classic antidepressants

Antidepressants have been used for a long time, but the effectiveness of antidepressants varies widely among individuals. To study the biological mechanisms behind this, Lukiæ et al. (2019) used three antidepressants (SSRI, SNRI, NRI) to treat depressed mice. They analyzed variations in GM by 16s rRNA gene sequencing. The results show that it all can alter the composition of the GM; only the specific strains and the degree of impact differ (Lukiæ et al., 2019; Sun et al., 2019). We hypothesized that the strength of antidepressant efficacy might be related to the ability to restore GM homeostasis.

Ketamine, the classic drug for treatment-resistant depression, substantial evidence has indicated that some of its antidepressant effects may be related to the restoration of GM (Hua et al., 2022). Huang et al. (2019) found that ketamine significantly improved the species diversity (α-diversity) of the GM of depressed mice. The β-diversity analysis also indicated a significantly different profile after ketamine administration (Huang et al., 2019). Yang et al. (2017) further investigated the two ketamine enantiomers and found that (R)-ketamine significantly reduced *Mollicutes* and butyricimonas levels in CSDS mice compared to (S)-ketamine. This may explain why (R)-ketamine has a greater and longer-lasting potency than (S)-ketamine. Other studies have shown that (R)-ketamine-mediated changes in GM are associated with the activation of microglia and bone mineral density, which is also inextricably linked to depression (Wan et al., 2022; Wang et al., 2022). Other first-line nervous systems drugs, such as sertraline, imipramine, and chlorpromazine, have also shown regulatory potential in these pathways in the GM. These drugs can significantly increase the levels of GABA and DA and reduce the glutamic acid metabolism of GM (Ma et al., 2021). The antibiotic rifaximin can also increase the relative abundance of *Ruminococci*, and *Spirillaceae*, increasing butyrate concentration in the brain (Li et al., 2021).

In addition to antidepressants modulating the abundance of specific strains, antidepressant efficacy is also influenced by GM. For example, *Adlercreutzia equolifaciens* can diminish the therapeutic effect of duloxetine (Lukiæ et al., 2019). It is important to note that the antibiotic-like effects of psychotropic drugs on GM are sometimes overlooked. Drugs inhibit the growth of GM, thereby inducing dysbiosis and causing disease progression, which may be an actual reason for the poor outcome of current clinical treatment (Ait Chait et al., 2021).

### Chinese materia medica

As early as the Han Dynasty in China, Zhang Zhongjing documented classical herbal formulas for the treatment of depression, but the specific pharmacology mechanism of the recipe is unclear (Chi et al., 2019). In recent studies, the antidepressant-like effects exerted by some CMM have been shown to correlate with GM. For example, Shugan Granule can significantly improve abnormal behavior and inflammation in the hippocampus of chronic restraint stress mice. Follow-up mechanistic studies have shown that its administration enriches *Butyricimonas* and *Candidatus Arthromitus* in the gut of mice, reduces the abundance of *Bacteroides*, and is closely associated with the PI3K/Akt/mTOR pathway (Li et al., 2022). Schisandra has demonstrated that it can attenuate gut dysbiosis in depressive mice by inhibiting TLR4/NF-κB signaling pathway (Sun et al., 2020). In addition to modulating inflammation, CMM also plays a role in regulating neurotransmitter levels. Neferine treatment can reduce the immobility time of depressed mice and increase the neurotransmitters in the hippocampus, such as DA, 5-HT, and NE. Meanwhile, an increase in the relative abundance of *Lactobacillus* in the mice colon was also observed (Dong et al., 2022). In another study, the targets of specific antidepressant CMM can focus on the regulation of purine metabolism (Ji et al., 2022). Animal studies related to CMM, such as cistanche tuberosa (Fan et al., 2021) and crocetin (Lin et al., 2021), have indicated that their antidepressant-like effects are closely related to the regulation of GM composition.

In addition to animal studies, clinical studies have provided corresponding evidence. Shao et al. (2021) interfered with the herbal formula Xiao-Chai-Hu-Tang for cancer patients with depression. After administration, depressive symptoms were lessened, and gut dysbiosis was partially reversed in subjects (particularly reducing abundances of *Parabacteroides*, *Blautia*, and *Ruminococcaceae bacterium*). Interestingly, this anti-depressive herbal medicine also exhibited some antitumor effects, with potential mechanisms involving the TLR4/MyD88/NF-κB signaling (Shao et al., 2021). Notably, these pathways also play an essential role in the pathogenesis of depression involving microbiota. Recent systematic reviews and meta-analyses have also shown that CMM reduces adverse events compared to antidepressants (Wang et al., 2019). Given the limited number of current studies, it is premature to summarize the benefits and risks of CMM for depression accurately.

### Dietary

Nutritional psychiatry supports the notion that dietary therapy can relieve depressive symptoms and promote brain health (Marx et al., 2021). At the same time, long-term dietary patterns also influence human GM (Bolte et al., 2021). Yan et al. (2021) found that maternal consumption of sulforaphane glucosinolate during pregnancy and lactation can affect the GM diversity of the offspring. This effect reduces the likelihood of stress-related psychiatric disorders in the offspring (Wei et al., 2022). In addition, specific types of dietary such as the Mediterranean diet (mainly fiber, fish, and whole grains), have been shown to modulate the GM composition by increasing the abundance of microorganisms that metabolize SCFAs, thereby reducing the onset of depression (Wei et al., 2022). Ketogenic diets (low carbohydrate, moderate protein, high fat) can help improve the efficiency of monoaminergic drugs by increasing GABA levels. And it also influences GM composition, such as an increased abundance of *Enterobacteriaceae*, *Akkermansia*, and *Slackia*, as well as a decreased abundance of *Bifidobacteria* and *Lachnobacterium* (Włodarczyk et al., 2021). An interventional randomized clinical trial demonstrated that flavonoid-rich orange juice could alleviate depression by increasing BDNF and the *Trichophyton* family (Ortega et al., 2022). Polyphenols have also been shown to ameliorate depressive symptoms by inhibiting the mitogen-activated protein kinase pathway involved in oxidative stress and inflammation (Behl et al., 2022). Polyphenols also act as prebiotics to provide nutrients for microorganisms, which complement each other (Zhou et al., 2020). A healthy dietary pattern will enhance the abundance of beneficial genera and thus reduce the risk of depression. We believe that future nutritional intervention studies also need to consider the regulation of GM as an essential part.

### Direct intervention in microbiota structure

#### Psychobiotics for depression, probiotics

Recent meta-analyses and systematic reviews have confirmed the antidepressant efficacy of probiotics in clinical studies (Amirani et al., 2020; Nikolova et al., 2021). Relevant animal studies have also shown that this effect is related to the regulation of the structure of the GM. Marin et al. (2017) used *Lactobacillus reuteri* to intervene in CUMS mice and found that only restoring *Lactobacillus* levels was sufficient to improve the metabolic changes and behavioral abnormalities associated with stress. In another study, Heat-sterilized *Bifidobacterium shortum* can modulate GM composition and thus prevent the depressive symptoms induced by chronic social defeat stress. Such strains with functional food components could be used as novel therapies (Kosuge et al., 2021; Tian et al., 2022). Of note, studies have shown that co-administration of probiotics and prebiotics can achieve a greater antidepressant effect (Moludi et al., 2022). Although some studies have found no significant improvement in depressive symptoms with prebiotics alone, it is possible that the substantial nutritional effect of prebiotics increases the survival rate of probiotics and thus amplifies the antidepressant ability of probiotics (Markowiak and Śliżewska, 2017; Alli et al., 2022; Karbownik et al., 2022). No relevant studies have reported problems such as withdrawal reactions and side effects caused by probiotics, which appear to be a promising intervention in depression. The optimal combination of probiotic strains, doses, and methods is not yet precise. To solve these problems, additional randomized, double-blind, placebo-controlled trials are needed to unravel the puzzle.

#### Fecal microbiota transplantation

Another way to directly interfere with GM is fecal microbiota transplantation (FMT). Its excellent performance in depression and microbial structure disorders has led us to see new treatment orientations (Bakken et al., 2011; Fond et al., 2020). In an animal study by Zhang et al. (2019), transplantation from *NLRP3 KO* flora significantly ameliorated depression-like behavior in recipient mice. In their inquiry, the FMT mechanism primarily relies on the inhibition of circHIPK2 expression in depressive mice. In human subjects, FMT also showed similar effects. In a case report of FMT as an adjunctive treatment for depression, the patients enrolled showed an improvement in depressive symptoms, an increase in GM diversity, and a relief in gastrointestinal symptoms. However, the effect was not long-lasting, and they speculated that the efficacy of FMT was related to the microbial similarity of the donor and the recipient (Doll et al., 2022). The main challenge for FMT is enhancing the success rate, the optimal delivery route, donor selection, and other issues that need to be explored. Our perceptions and insights are still growing.

Recently, a novel type of transplant that is also an alternative to FMT, Microbial Ecosystem Therapeutic (MET)-2, demonstrated good antidepressant results in phase 2, a double-blind, placebo-controlled study. An increase in the abundance and diversity of beneficial bacteria was observed in the MET-2 group gut, suggesting that the use of MET-2 may promote the repopulation of the GM and modulate the healthy genus associated with MGBA (Chinna Meyyappan et al., 2021). However, the study’s sample size was too small, and a larger multicenter trial is needed to validate the feasibility of MET-2. In addition, Liu S. et al. (2016) showed that fecal microRNA could regulate inter-species genes to facilitate the host GM. We speculate that transplantation of depression-related miRNA (has-miR-139-3p, miRNAs-let-7e-5p, has-let-7f-5p, etc.) restored to their normal levels may also have a therapeutic effect, which requires further animal and clinical studies to verify (Gecys et al., 2022).

## Summary and perspective

In summary, depression can alter the composition of the GM, and gut dysbiosis can further exacerbate depression’s severity. This tight inter-regulatory link has increased the possibility of alleviating depression by regulating GM. Therefore, we have summarized and integrated current studies on possible pathophysiological mechanisms of GM involved in depression and exemplified existing alleviating regimens related to GM. We hope to provide some reference for future exploration of microbiota targeting measures.

In future research, we need to standardize assessing depression degree, which is related to the accuracy and reproducibility of experimental results of depression-related mechanisms. Notably, more longitudinal and human studies are required to fill in the gaps in current knowledge. In addition, understanding the dynamics of microbial metabolites *in vivo* remains a significant challenge. As more studies are conducted, it is expected that the pathogenesis of depression will be further elucidated in the future, laying the foundation for developing microbiome-based therapies.

## Author contributions

JX and TS conceived and designed the review. WH and JX wrote the draft of manuscript. MH and NW collected the information. MB worked on the electronic manuscript of all the figures in this review. All authors read and approved the final manuscript and submission of this manuscript.
